# Supplementary material for: Reclassifying lethal heat
Source: Nat Commun. 2026 Apr 3;17:4801. doi: 10.1038/s41467-026-71396-x (PMC13219605; doi:10.1038/s41467-026-71396-x)
Supplement: Supplementary file 1 — Supplementary Information [file 41467_2026_71396_MOESM1_ESM.pdf]

Supplementary S.1 Sampling Strategy & Hyperparameter Tuning

We investigate the training set composition and sampling strategy by increasing the ratio of lethal to nonlethal heatwave events in the training set by decreasing numbers of nonlethal events in the training set accordingly, implementing a down-sampling experiment. As described in the Methods, for all experiments, we utilised the same training, validation, and test set splits, taking the most recent 10% of events by region as the test set. We selected a further 10% of events from the training set without stratification to use as the validation set for model optimisation, thereby creating a split of 80%, 10%, and 10% across the training, validation, and test sets, respectively. Any events removed through downsampling are not added to the validation or test set. We set the hyperparameters for the number of trees and maximum depth of those trees arbitrarily to $n_{trees} = 128$ and $d_{max} = 32$. The results for this are shown in Supplementary Figure 1.

Varying the training set composition adjusts the balance between recall, precision, and F1 scores, with a higher proportion of the training set being lethal events leading to higher recall but a lower precision. Decreasing the proportion of lethal events leads to the converse. Four scenarios from the 100 different sampling regimes are extracted and presented in Supplementary Table 1, with scenarios labelled according to the proportion of lethal events in the training set achieved via downsampling the nonlethal events, along with performance achieved using SMOTE.

SMOTE and downsampling to a 1:1 ratio of lethal to nonlethal events result in a similar effect on the feature distributions, as shown in Supplementary Figures 2 and 3, due to both increasing the relative proportion of lethal events to the same amount. In both cases, the marginalised feature distributions for the lethal and nonlethal events are essentially unchanged from the original distributions. Whilst the feature distributions

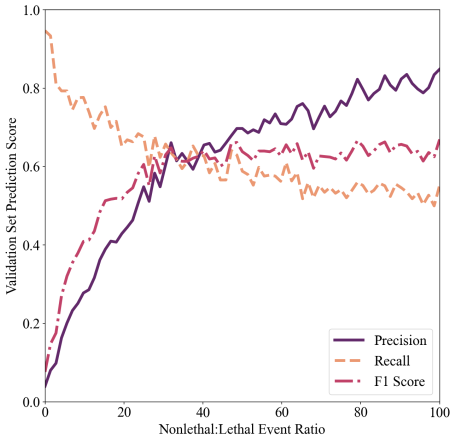

Supplementary Fig. 1 Model performance on the validation set with respect to training set downsampling ratio. Precision, Recall, and F1 scores on training and validation set prediction performance with respect to proportion of nonlethal to lethal (n:1 where n is the value on the x axis) events within the training set.

Supplementary Table 1 Performance metrics of lethal/nonlethal heatwave classification for different training set compositions achieved via downsampling and using SMOTE.

Sampling	Metric			
	Accuracy Score	Precision	Recall	F1 Score
50% Lethal	0.972	0.040	0.946	0.077
10% Lethal	0.989	0.251	0.775	0.379
2% Lethal	0.994	0.697	0.629	0.661
1% Lethal	0.995	0.834	0.532	0.650
SMOTE	0.996	0.779	0.699	0.737

are relatively similar between the two different strategies, the lower number of permutations from the downsampled distributions reduces the model performance in terms of precision and F1 score.

SMOTE was therefore selected as the strategy for redressing class imbalance based on the overall best F1 score. We then optimise the model hyperparameters by running a search procedure, searching through 10,000 possible combinations for number of decision trees, $1 < n_{trees} < 200$, and maximum tree depth, $1 < d_{max} < 50$. A subset of the results from this procedure are shown in Supplementary Figure 4.

From the hyperparameter optimisation, increasing the number of trees and the maximum depth improves model performance, converging at $n_{trees} \approx 20$ and $d_{max} \approx 33$. The nature of convergence for varying maximum depth suggests that this hyperparameter could be used to achieve higher recall at the expense of precision in a more optimal way than downsampling.

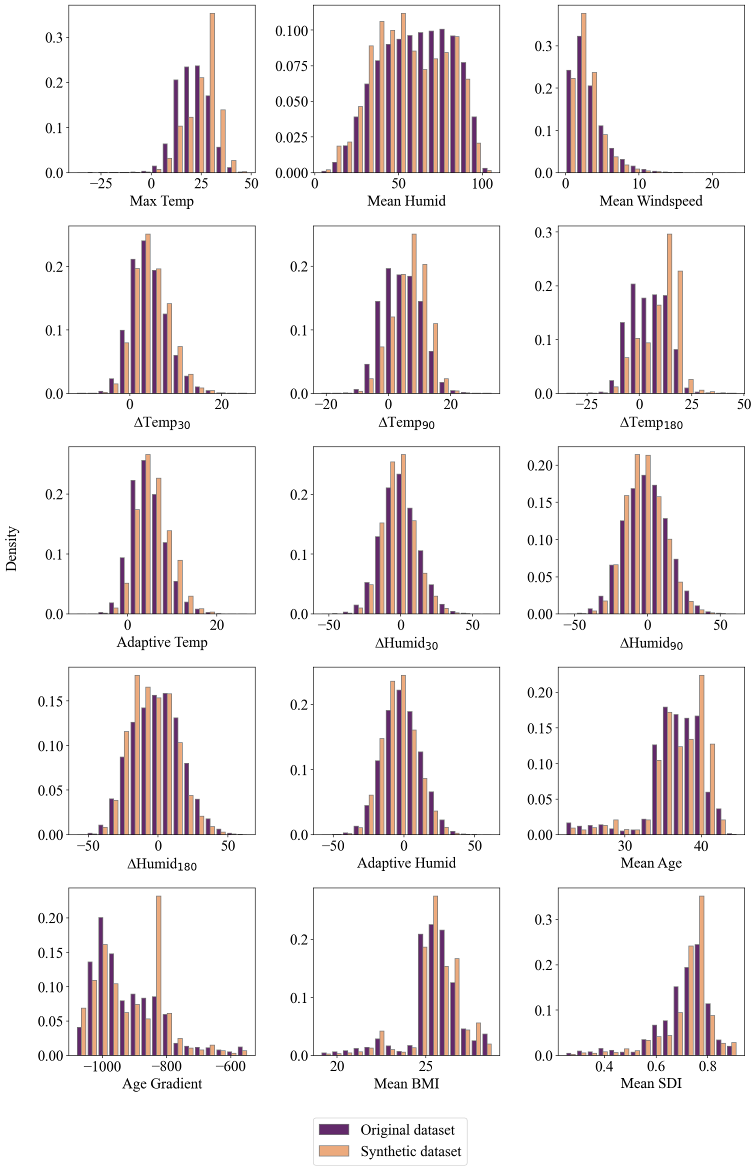

Supplementary Fig. 2 Feature distributions for the original training data and adjusted training data using SMOTE. Histograms to represent the feature distributions across all input features for the unaltered and synthetic data sets, taken from the validation subset of each, where: Max Temp is the maximum temperature, Mean Humid is the mean relative humidity, ΔT_{30} is the temperature differential over the previous 30 days' average, ΔT_{90} is the temperature differential over the previous 90 days' average, ΔT_{180} is the temperature differential over the previous 180 days' average, ΔH_{30} is the humidity differential over the previous 30 days' average, ΔH_{90} is the humidity differential over the previous 90 days' average, ΔH_{180} is the humidity differential over the previous 180 days' average, Age Gradient is the slope of the population pyramid, Mean BMI is the average Body Mass Index across the whole population, and Mean SDI is the average Socio-Demographic Index. The unaltered validation dataset contains 11,287 members. The synthetic validation dataset contains 22,398 members.

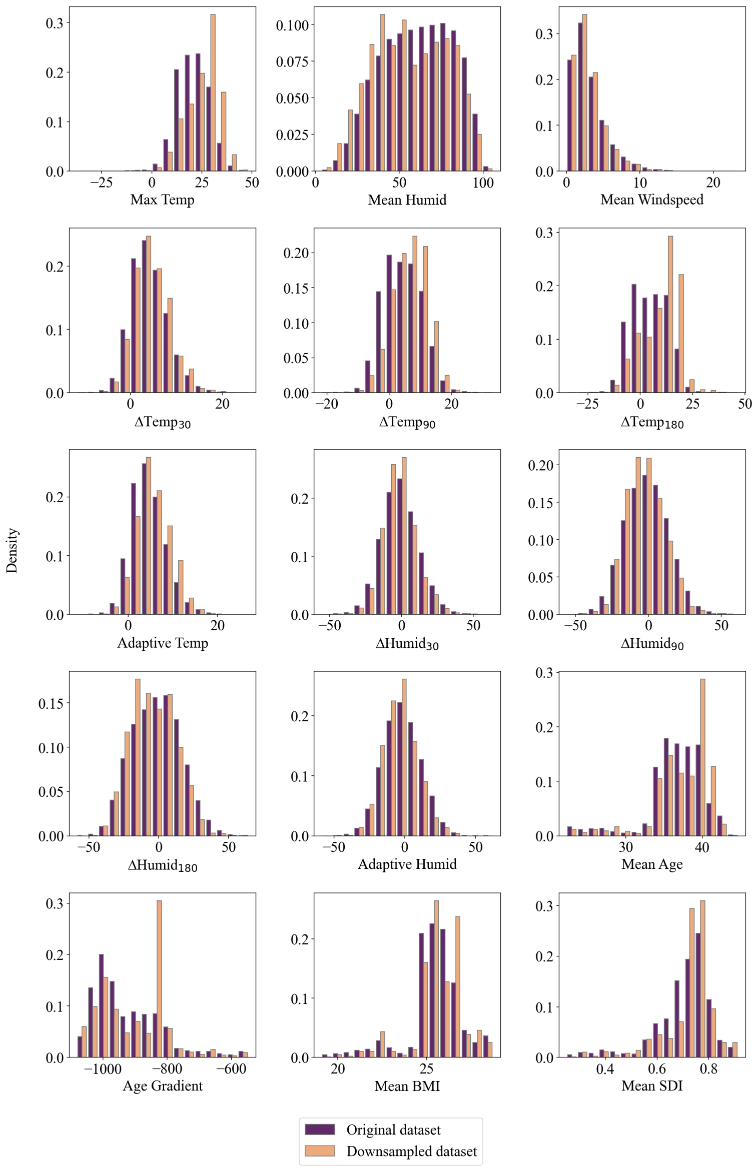

Supplementary Fig. 3 Feature distributions for the original training data and adjusted training data using downsampling. Histograms to represent the feature distributions across all input features for the unaltered and downsampled data sets, taken from the validation subset of each, where: Max Temp is the maximum temperature, Mean Humid is the mean relative humidity, ΔT_{30} is the temperature differential over the previous 30 days' average, ΔT_{90} is the temperature differential over the previous 90 days' average, ΔT_{180} is the temperature differential over the previous 180 days' average, ΔH_{30} is the humidity differential over the previous 30 days' average, ΔH_{90} is the humidity differential over the previous 90 days' average, ΔH_{180} is the humidity differential over the previous 180 days' average, Age Gradient is the slope of the population pyramid, Mean BMI is the average Body Mass Index across the whole population, and Mean SDI is the average Socio-Demographic Index. The unaltered validation dataset contains 11,287 members. The downsampled validation dataset contains 176 members.

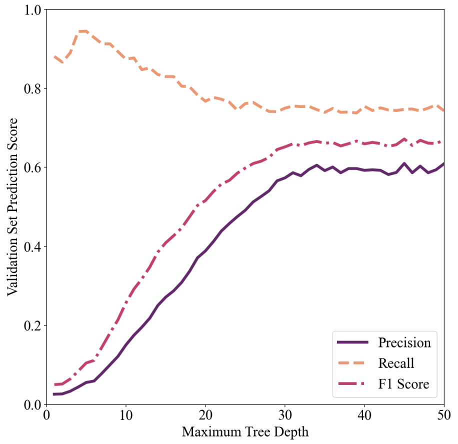

(a) Validation set performance under varying number of decision tree classifiers for maximum depth fixed at $d_{max} = 32$

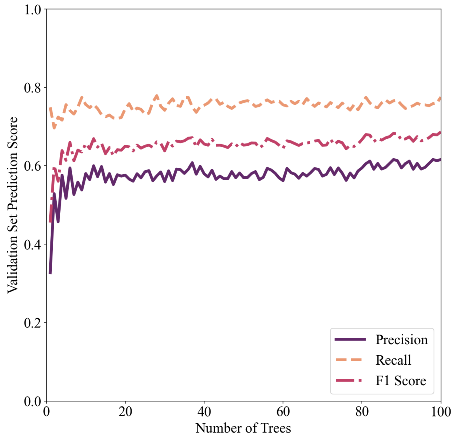

(b) Validation set performance under varying maximum tree depth for number of decision tree classifiers fixed at $n_{trees} = 128$

Supplementary Fig. 4 Model performance on the validation set for varying hyperparameters. Precision, Recall, and F1 scores on validation set performance with respect to a subset of combinations from the hyperparameter optimisation process, covering tree depth and number of trees.
