# Supplementary material for: Reclassifying lethal heat
Source: Nat Commun. 2026 Apr 3;17:4801. doi: 10.1038/s41467-026-71396-x (PMC13219605; doi:10.1038/s41467-026-71396-x)
Supplement: Supplementary file 2 — Reporting Summary [file 41467_2026_71396_MOESM2_ESM.pdf]

Corresponding author(s): Robert Edwin Rouse

Last updated by author(s): 08/02/2026

Reporting Summary

Nature Portfolio wishes to improve the reproducibility of the work that we publish. This form provides structure for consistency and transparency in reporting. For further information on Nature Portfolio policies, see our Editorial Policies and the Editorial Policy Checklist.

Statistics

For all statistical analyses, confirm that the following items are present in the figure legend, table legend, main text, or Methods section.

n/a	Confirmed	
☐	☒	The exact sample size (n) for each experimental group/condition, given as a discrete number and unit of measurement
☒	☐	A statement on whether measurements were taken from distinct samples or whether the same sample was measured repeatedly
☒	☐	The statistical test(s) used AND whether they are one- or two-sided<br>*Only common tests should be described solely by name; describe more complex techniques in the Methods section.*
☒	☐	A description of all covariates tested
☒	☐	A description of any assumptions or corrections, such as tests of normality and adjustment for multiple comparisons
☐	☒	A full description of the statistical parameters including central tendency (e.g. means) or other basic estimates (e.g. regression coefficient) AND variation (e.g. standard deviation) or associated estimates of uncertainty (e.g. confidence intervals)
☒	☐	For null hypothesis testing, the test statistic (e.g. F, t, r) with confidence intervals, effect sizes, degrees of freedom and P value noted<br>*Give P values as exact values whenever suitable.*
☒	☐	For Bayesian analysis, information on the choice of priors and Markov chain Monte Carlo settings
☒	☐	For hierarchical and complex designs, identification of the appropriate level for tests and full reporting of outcomes
☒	☐	Estimates of effect sizes (e.g. Cohen's d, Pearson's r), indicating how they were calculated

Our web collection on statistics for biologists contains articles on many of the points above.

Software and code

Policy information about availability of computer code

Data collection	Data was collected using a combination of direct download of data sources for which no API existed and APIs, where applicable. The latter includes the CDS API. The UN population API was publicly available but is now deprecated. All code required to download the data and links for where no API is available is included in the codebase for this study, available in the following GitHub repository: https://github.com/robert-edwin-rouse/reclassifying-lethal-heat.
Data analysis	The code used to produce the results of the study is available in the following GitHub repository: https://github.com/robert-edwin-rouse/reclassifying-lethal-heat. This codebase is written in Python 3.13.0 and makes use of the following libraries: cartopy 0.22.0, cdsapi 0.7.4, country_converter 1.3.1, geopandas 1.0.1, geopy 2.4.1, imblearn 0.12.3, matplotlib 3.8.4, numpy 1.25.2, pandas 2.3.1, requests 2.32.4, scikit-learn1.3.0, scipy 1.11.4, seaborn 0.13.2, xarray 2023.6.0.

For manuscripts utilizing custom algorithms or software that are central to the research but not yet described in published literature, software must be made available to editors and reviewers. We strongly encourage code deposition in a community repository (e.g. GitHub). See the Nature Portfolio guidelines for submitting code & software for further information.

Data

Policy information about availability of data

All manuscripts must include a data availability statement. This statement should provide the following information, where applicable:

- Accession codes, unique identifiers, or web links for publicly available datasets
- A description of any restrictions on data availability
- For clinical datasets or third party data, please ensure that the statement adheres to our policy

The following publicly available data was used in this study: the ECMWF's ERA5 reanalysis product, which can be obtained from https://cds.climate.copernicus.eu/cdsapp#!/dataset/reanalysis-era5-pressure-levels; the UN's population data, which can be obtained from https://population.un.org/wpp/; the NCD Risk Factor Collaboration's BMI data, which can be obtained from the Lancet via https://doi.org/10.5281/zenodo.10534960; and the Global Burden of Disease Study 2021 (GBD 2021) Socio-Demographic Index (SDI) 1950–2021, obtainable from the Global Health Data Exchange via https://doi.org/10.6069/dwqg-3z75. The heatwave lethality data was obtained by contacting the authors of the following paper: https://doi.org/10.1038/nclimate3322; the data owners will make this data available through a public repository upon acceptance of this paper.

Research involving human participants, their data, or biological material

Policy information about studies with human participants or human data. See also policy information about sex, gender (identity/presentation), and sexual orientation and race, ethnicity and racism.

Reporting on sex and gender	N/A
Reporting on race, ethnicity, or other socially relevant groupings	N/A
Population characteristics	N/A
Recruitment	N/A
Ethics oversight	N/A

Note that full information on the approval of the study protocol must also be provided in the manuscript.

Field-specific reporting

Please select the one below that is the best fit for your research. If you are not sure, read the appropriate sections before making your selection.

☐ Life sciences ☐ Behavioural & social sciences ☒ Ecological, evolutionary & environmental sciences

For a reference copy of the document with all sections, see nature.com/documents/nr-reporting-summary-flat.pdf

Ecological, evolutionary & environmental sciences study design

All studies must disclose on these points even when the disclosure is negative.

Study description	We used a random forest model to create a classifier for determining heatwave lethality or non-lethality based on a dataset comprising 125,411 events where the temperature exceeded the ninetieth percentile across 140 cities, with combined meteorology and sociodemographic inputs to label these events. The accuracy of our model outperformed classification that relied on wet bulb temperature thresholds with a factor of 11 improvement in imbalanced classification performance. Furthermore, we found that the majority of lethal heatwaves within our dataset occur below high wet bulb temperature thresholds and that accurate predictions for heatwave mortality could be obtained by combining thermo-temporal differentials and population health metrics instead of absolute climatic conditions.
Research sample	The research sample used here is the documented heatwave events from the dataset collected in Global risk of deadly heat, Mora et al, Published: 19 June 2017 in Nature Climate Change (https://doi.org/10.1038/nclimate3322) that exceed the ninetieth temperature percentile, comprising 125,411 heatwave events occurring in 140 cities around the world. This was combined with United Nations data on population pyramids, Body Mass Index, Global Burden of Disease Socio-Demographic Index, and climatic data taken from the ECMWF's ERA5 reanalysis data product. This heatwave data was utilised due to it being a comprehensive dataset on the time and location of lethal/non-lethal heatwaves. The other data sources were utilised due to their ubiquity in terms of matching the temporal and spatial range of the heatwave dataset, where no other data sets have the same spatio-temporal characteristics and are therefore unsuitable.
Sampling strategy	The heatwave data was collected from 1980 to 2014 with a geographic span over 164 cities across 36 countries. Public health and sociodemographic data was taken from studies conducted at the national scale, unadjusted for subnational regions. There are approximately 979 lethal heatwaves with the rest being nonlethal; as a percentage, 0.78% are lethal and 99.2% are nonlethal. The full dataset was utilised across the training, validation, and test sets to maximise representation within the training set and was split

	as follows: 10% was taken as the test set through a chronological splitting method to minimise temporal leakage and the remaining 90% split randomly into the training and validation sets for a 80/10/10 data set split.
Data collection	The heatwave lethality data was collected by the authors of the paper, Global risk of deadly heat, Mora et al, Published: 19 June 2017 in Nature Climate Change (https://doi.org/10.1038/nclimate3322). In this work, the authors reviewed 30,000 references, spanning 783 cases from 164 cities across 36 countries, between 1980 and 2014 and documented cases of excess human mortality associated with heat. The lethal/non-lethal heatwave labels have been made available by Dousset and contributors through the following repository https://doi.org/10.5281/zenodo.18528487. The following publicly available data was used in this study: the ECMWF's ERA5 reanalysis product, which can be obtained from https://cds.climate.copernicus.eu/cdsapp#!/dataset/reanalysis-era5-pressure-levels; the UN's population data, which can be obtained from https://population.un.org/wpp/; the NCD Risk Factor Collaboration's BMI data, which can be obtained from the Lancet via https://doi.org/10.5281/zenodo.10534960; and the Global Burden of Disease Study 2021 (GBD 2021) Socio-Demographic Index (SDI) 1950–2021, obtainable from the Global Health Data Exchange via https://doi.org/10.6069/dwqg-3z75.
Timing and spatial scale	The heatwave data were collected from 1980 to 2014 with a geographic span over 164 cities across 36 countries. Meteorological data were taken for a 1° x 1° latitude longitude grid square around each city from 1970 to 2014 at an hourly resolution. The Lancet BMI, GBD SDI, and UN population data were taken from studies conducted at the national scale, unadjusted for subnational regions, for 1980 to 2014 at yearly resolution.
Data exclusions	No data was excluded in this study.
Reproducibility	The code, including that which installs open source dependencies, to reproduce all results from this study is available at https://github.com/robert-edwin-rouse/reclassifying-lethal-heat. The data will be made available upon acceptance. No other materials are required.
Randomization	Randomisation was not relevant to this study because we didn't allocate samples/organisms/participants into experimental groups and therefore was not conducted.
Blinding	Blinding was not relevant to this study because we didn't allocate samples/organisms/participants into experimental groups and therefore was not conducted.

Did the study involve field work? ☐ Yes ☒ No

Reporting for specific materials, systems and methods

We require information from authors about some types of materials, experimental systems and methods used in many studies. Here, indicate whether each material, system or method listed is relevant to your study. If you are not sure if a list item applies to your research, read the appropriate section before selecting a response.

Materials & experimental systems

n/a	Involved in the study
☒	☐ Antibodies
☒	☐ Eukaryotic cell lines
☒	☐ Palaeontology and archaeology
☒	☐ Animals and other organisms
☒	☐ Clinical data
☒	☐ Dual use research of concern
☒	☐ Plants

Methods

n/a	Involved in the study
☒	☐ ChIP-seq
☒	☐ Flow cytometry
☒	☐ MRI-based neuroimaging

Plants

Seed stocks	N/A
Novel plant genotypes	N/A
Authentication	N/A
